# Klotho Alleviates Lung Injury Caused by Paraquat via Suppressing ROS/P38 MAPK-Regulated Inflammatory Responses and Apoptosis

**DOI:** 10.1155/2020/1854206

**Published:** 2020-05-13

**Authors:** Zhiqiang Zhang, Qing Nian, Gang Chen, Shuqing Cui, Yuzhen Han, Jinying Zhang

**Affiliations:** ^1^Department of Emergency, Binzhou Medical University Hospital, Binzhou 256603, China; ^2^Department of Oncology, Hospital of Chengdu University of Traditional Chinese Medicine, Chengdu 610072, China; ^3^Department of Vascular Intervention, Binzhou Medical University Hospital, Binzhou 256603, China; ^4^Standardized Residency Training Center, Binzhou Medical University Hospital, Binzhou 256603, China; ^5^Department of Pathology, Binzhou Medical University Hospital, Binzhou 256603, China

## Abstract

Acute lung injury (ALI) induced by paraquat (PQ) progresses rapidly with high mortality; however, there is no effective treatment, and the specific mechanism is not well understood. The antiaging protein klotho (KL) has multiple functions and exerts significant influences on various pathophysiological processes. This work evaluated the impact of KL on PQ-induced ALI and investigated its underlying mechanisms. As for *in vivo* research, C57BL/6 mice were treated with PQ (30 mg/kg) intraperitoneal (IP) injection to create a toxicity model of ALI (PQ group). The mice were divided into control group, KL group, PQ group, and PQ+KL group. For *in vitro* experiment, A549 cells were incubated with or without KL and then treated in the presence or absence of PQ for 24 h. *In vivo* result indicated that KL reduced the mortality, reduced IL-1*β* and IL-6 in the bronchoalveolar lavage fluid (BALF), attenuated ALI, and decreased apoptosis *in situ*. *In vitro* result revealed that KL significantly improved cell viability, reduced the levels of IL-1*β* and IL-6 in culture supernatants, suppressed cell apoptosis, inhibited caspase-3 activation, and enhanced mitochondrial membrane potential (*ΔΨ*m) after PQ treatment. Besides, KL effectively abated reactive oxygen species (ROS) production, improved GSH content, and lowered lipid peroxidation in PQ-exposed A549 cells. Further experiments indicated that phosphorylated JNK and P38 MAPK was increased after PQ treatment; however, KL pretreatment could significantly lower the phosphorylation of P38 MAPK. Suppression of P38 MAPK improved cell viability, alleviated inflammatory response, and reduced apoptosis-related signals; however, it had no obvious effect on the production of ROS. Treatment with N-acetylcysteine (NAC), a classic ROS scavenger, could suppress ROS production and P38 MAPK activation. These findings suggested that KL could alleviate PQ-caused ALI via inhibiting ROS/P38 MAPK signaling-regulated inflammatory responses and mitochondria-dependent apoptosis.

## 1. Introduction

Paraquat (PQ), which is a quaternary nitrogen herbicide, is extensively utilized in agriculture for weed control. Over the last several decades, PQ has been broadly used worldwide, particularly in Asia, due to its quick action, broad spectrum of activity, and relatively low cost [1]. However, PQ is highly toxic to animals and humans by direct contact. PQ can enter the human body through the skin, digestive tract, and respiratory tract. In recent years, the prevalence of PQ intoxication still remained high, and the mortality rate was over 90% [2]. After ingestion, PQ distributes in multiple organs, such as kidneys, lungs, heart, liver, and gastrointestinal tract, leading to multiple organ dysfunction [3]. In the above organs, the lungs are the mainly affected organ [4]. Alveolar epithelial cells can actively absorb and accumulate PQ through the highly developed polyamine uptake system. The enrichment of PQ in alveolar epithelial cells could lead to alveolar epithelial rupture, impaired surfactant production, hemorrhage, edema, and inflammatory cell infiltration, which leads to ALI and progressive respiratory failure [5].

So far, most of the research findings indicated that PQ exerts the toxic effect via sustained redox-cycling reactions, resulting in overproduction of ROS [6]. Excessive accumulation of intracellular ROS can lead to oxidative stress-induced injuries to cellular organelles, nucleic acids, proteins, and lipids [7, 8]. ROS also functions as key regulators of cell signaling involved in the regulation of cell growth, development, inflammation, and apoptosis [9, 10]. Currently, there are no specific antidotes for PQ intoxication and the mortality remains very high. Because PQ toxicity is mainly related to the mechanism of oxidative stress, antioxidant therapy might be an effective way to cure PQ intoxication.

KL protein was originally discovered in mice and identified as the aging-suppressor gene. Previous findings have indicated that the secreted form of KL protein can function as circulating hormones to exert multiple physiological functions on target organs [11]. Furthermore, recent discoveries showed that KL has the effect of inhibiting ROS production and scavenging the overproduced ROS in mouse tubular epithelial cells [12]. Previous study demonstrated that KL protein administration could facilitate removal of ROS and increase resistance to oxidative stress in Hela and CHO cells receiving PQ treatment [13]. Ravikumar et al. reported that exogenous or endogenous KL protein could attenuate hyperoxia- and phosphotoxicity-induced oxidative damage in pulmonary epithelia [14]. Similarly, an *in vitro* experiment demonstrated that increasing KL expression in lung epithelial cells reduced cigarette smoke extract-induced cell death by decreasing intracellular ROS production and suppressing cellular senescence [15]. Based on the above findings, we hypothesized that administration of KL protein could attenuate PQ-caused pulmonary injury and dysfunction via reducing intracellular ROS production. To test this hypothesis, we performed both *in vitro* and *in vivo* experiments to determine whether KL protein exerts protective effects on oxidative injuries in lung epithelial cells and try to clarify the relevant mechanisms involved in this process.

## 2. Materials and Methods

### 2.1. Rodent

Healthy male C57BL/6 mice (8-10 weeks) were obtained from the Experimental Animals Center of Binzhou Medical University Hospital (Binzhou, China). The mice had free access to sterile water or food. After a 7-day adaptive period in an animal facility without specific pathogens, mice in good health were chosen to use. Mice were anesthetized by IP injecting 1% pentobarbital sodium (50 mg/kg). The animal experiment was approved by the Animal Ethics Committee of Binzhou Medical University Hospital.

### 2.2. Experimental Design

PQ dichloride (Sigma-Aldrich, USA) was dissolved in distilled water. For PQ treatment, the mice were IP administered PQ at a single dose of 30 mg/kg. The PQ dosage was selected according to former studies [16, 17] and our preliminary experiments. An identical volume of normal saline was applied IP as control. For KL administration, the recombinant mouse KL protein (R&D Systems, USA; 10 *μ*g/kg) dissolved in phosphate-buffered saline (PBS, 0.01 mM) was injected IP 6 h prior to PQ treatment followed by every other day injection for 7 days. The dosage of KL was selected based on previous studies [18]. The mice were randomly assigned to four groups of 12 and studied concomitantly. The grouping design is as follows: control group (mice receiving IP injection of PBS), KL group (mice receiving IP injection of KL), PQ group (mice receiving PQ treatment), and KL+PQ group (mice receiving IP injection of KL 6 h before PQ treatment). The mice were sacrificed concurrently 7 days after PQ administration.

### 2.3. Bronchoalveolar Lavage

The collection of BALF was performed based on the previous study [19]. The mice were anaesthetized and sacrificed seven days post PQ treatment. Thoracotomy was performed immediately after disinfection, and the right major bronchial beneath the tracheal bifurcation was ligated. Then, the left bronchus was cannulated and flushed with ice-cold PBS (0.5 mL) three times in triplicate. Subsequently, the collected BALF was centrifuged at 1000g for 10 min at 4°C, then the supernatant was harvested and stored in -80°C for further detection.

### 2.4. Ratios of Lung Wet-to-Dry Weight (W/D)

The middle lobe of the right lung was isolated, and the wet weight was measured after blood and surface moisture was blotted off. Next, the lung tissue was heated at 70°C for 72 h to stabilize the dry weight. Then, the ratios of W/D were calculated to evaluate lung tissue edema.

### 2.5. Histopathological Evaluations

For histopathological assessments, the lungs were removed and fixed with 10% formalin, paraffin-embedded and sliced at 5 *μ*m. Hematoxylin and eosin staining was used for light microscopic examination. Lung injury was scored based on the methods as previously reported [20, 21]. The histopathologic parameters used for scoring include alveolar congestion, hemorrhage, neutrophil infiltration, and thickening of the alveolar wall. Severity of injuries for each item was graded on a 5-point scale: 0 = little, 1 = mild, 2 = moderate, 3 = severe, and 4 = maximal. The total injury score was calculated as the sum of the individual scores from each category.

### 2.6. TUNEL Assay

To detect apoptosis in situ of lung tissues, we used a terminal deoxynucleotidyl transferase-mediated biotinylated UTP nick end labeling (TUNEL) kit as our previous studies reported [22]. Briefly, lung tissue sections were dewaxed, rehydrated, and treated with proteinase K working solution for 30 min. Then, slides were kept in a TUNEL reaction mixture at 37°C for 1 h. After incubated with converter-POD, tissues were reacted with DAB substrate for 10 min followed by counterstaining with hematoxylin.

### 2.7. Cell Culture and Treatment

A549 cells were cultured in Dulbecco's Modified Eagle Medium (HyClone, USA) with 10% fetal bovine serum (Gibco, USA) at 37°C with 5% CO_2_. To measure PQ toxicity, A549 cells were exposed to different concentrations of PQ (0, 10, 20, 50, 100, 200, 500, and 1000 *μ*M) at different time periods (Fig. S1). A549 cell survival showed a dose- and time-dependent decrease at 0-1000 *μ*M and at 24-72 h, so a dose of 200 *μ*M and an incubating time of 24 h were selected in subsequent experiments. An identical volume of PBS was used as control.

To investigate whether KL administration could affect the cytotoxicity induced by PQ, A549 cell was preincubated with various doses (50-400 pM) of recombinant human KL protein (R&D Systems, USA) for 1 h followed by exposure to PQ for 24 h. For experimental treatments, A549 cells were grouped as follows: control group (PBS treatment only), KL group (KL preincubation only), PQ group (PQ treatment only), and PQ+KL group (KL preincubation followed by PQ treatment).

### 2.8. Cell Viability Assay

Cell viability was evaluated by a Cell Counting Kit-8 (Beyotime, China) as we previously described [22]. The absorbance of each sample was measured by a microplate reader (SpectraMax, USA) at 450 nm. Cell viability was showed as the ratio of absorbance to that in the control.

To further study the impact of KL on PQ-exposed A549 cells, Calcein-AM and propidium iodide (PI) double staining was used to detect living and dead cells. Calcein-AM was used for labeling of living cells, while PI for staining of dead cells. After incubation with the staining solutions for 15 min at 37°C, cells were observed by a fluorescence microscope (Olympus DP80, Japan).

### 2.9. ROS Production

The contents of intracellular ROS were detected by a ROS Assay Kit (Beyotime). Briefly, the DCFH-DA fluorescent probe was loaded to treated cells (5 × 10^4^ cells/mL) followed by incubation for 20 min at 37°C. DCFH-DA enters A549 cells and deacetylates to nonfluorescent DCFH by cellular esterases. Intracellular DCFH can be oxidized by ROS and produces the highly fluorescent DCF. The DCF fluorescence was observed using a fluorescence microscope (Olympus DP80, Japan).

### 2.10. Determination Levels of MDA, GSH, and Inflammatory Cytokines

The malondialdehyde (MDA) content was used as an indicator for lipid peroxidation. The intracellular MDA and GSH contents were detected using thiobarbituric acid and DTNB method, respectively. The value of MDA or GSH was calculated according to the equations of the manufacturer's directions (Nanjing Jiancheng Bioengineering Institute, China). IL-1*β* or IL-6 in BALF and culture supernatants was measured by ELISA kits (R&D Systems, USA) following the manufacturer's directions.

### 2.11. Detection Apoptosis with Flow Cytometry

A549 cell apoptosis was detected using the Annexin V-FITC/PI staining method. Briefly, A549 cells (approximately 1 × 10^6^/tube) were trypsinized, rinsed, and resuspended in 100 *μ*L of staining solution, then treated with Annexin V-FITC/PI solution at room temperature for 15 min. Finally, treated cell samples were analyzed by a BD Accuri C6 flow cytometer.

### 2.12. Measurement of *ΔΨ*m

The *ΔΨ*m was evaluated using a *ΔΨ*m assay kit with a JC-1 fluorescent probe (Beyotime). A549 cells were incubated with or without KL for 1 h and then treated with or without PQ (200 *μ*M) for 24 h. The cells were then treated with JC-1 at 37°C for 20 min and rinsed twice with JC-1 staining buffer. The JC-1 fluorescence was detected by a laser confocal microscope. The transition of JC-1 from red fluorescence to green fluorescence reflects the decrease in *ΔΨ*m. In the present work, the ratios of green to red fluorescence were calculated to assess mitochondrial depolarization.

### 2.13. Analysis of Caspase-3 Activity

Caspase activities were detected using a Caspase Activity Assay Kit (Beyotime). Briefly, cells were harvested, lysed, and centrifuged to collect the lysate supernatant. Subsequently, the total protein content was determined by the Bradford method. Finally, assay buffer, lysate supernatant, and Ac-DEVD-*p*NA were successively loaded on 96-well plates and incubated for 2 h at 37°C. The absorbance at 405 nm was detected by a microplate reader; then, the activity of caspase-3 was determined according to the *p*NA standard curve.

### 2.14. siRNA Transfection

To downregulate p38 MAPK, A549 cells were seeded on 6-well plates and transiently transfected with p38 MAPK siRNA (GenePharma, China) or nonspecific siRNA by Lipofectamine 2000 (Invitrogen, USA). After 24 h, cells were harvested and western blotting assay was used to assess the knockdown efficiency.

### 2.15. Western Blotting Analysis

Total cellular proteins of each sample were separated by SDS-PAGE electrophoresis, and western blots were conducted as our previous study described [22]. The proteins were transferred onto polyvinylidene fluoride membranes. After blocking, the membranes were overlaid with primary antibodies for phosphorylated or total p38, JNK, and ERK (1 : 500) overnight at 4°C, then incubated with secondary antibody at 37°C for 1 h. Proteins were visualized by enhanced chemiluminescence detection reagents (Beyotime). *β*-Actin was used as the endogenous control.

### 2.16. Statistical Analysis

The results were presented as mean ± standard deviation. Error bar represents standard deviations of three separate experiments. Differences between groups were compared using one-way ANOVA and Tukey's HSD test for multiple comparisons. A value was considered statistically significant when *P* < 0.05.

## 3. Results

### 3.1. KL Administration Prolonged Survival Rates of the ALI Mouse Model

**Figure 1:**
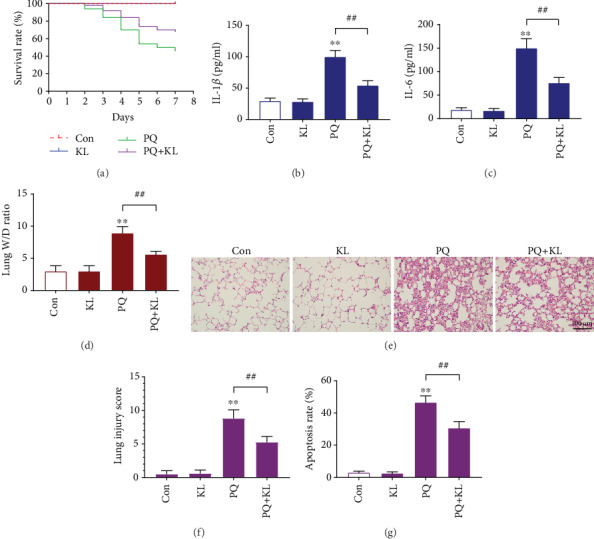
KL administration improves the survival rate and attenuates PQ-induced lung injury in the ALI mouse model. (a) Kaplan-Meier survival curve of rats receiving different treatments. KL administration improved the survival rate of rats receiving PQ treatment. The contents of the proinflammatory cytokines IL-1*β* (b) and IL-6 (c) in the BALF were measured by ELISA. (d) The lung W/D ratio was calculated to assess lung edema in different treatment groups. (e) H&E staining of lung tissue sections from each group of mice. This figure showed KL administration attenuated pathological damage after PQ application. (f) The lung injury scores for the lung tissue of different treatment groups. (g) Effects of KL administration on PQ- induced apoptosis of lung cells. Apoptosis in situ was measured using TUNEL assay. Data are shown as the mean ± SD. ^∗∗^*P* < 0.01 versus control group; ^##^*P* < 0.01 versus PQ group, ANOVA.

The mice in the PQ group exhibited decreased appetite and an increased respiratory rate after 24 h of PQ administration. Lip cyanosis, tachypnea, and nasal hemorrhage were observed from 24 to 48 h after PQ treatment. At 7 days post PQ treatment, the survival rates of each group displayed significant differences, with the PQ group being the lowest (Figure 1(a)). The survival rate of the KL group was 100%, indicating that KL administration had no obvious deleterious effects. The KL+PQ group had a higher survival rate compared with the PQ group, which indicated that KL administration reduced PQ-caused injuries. These results suggested that the *in vivo* administration of KL had a protective effect on ALI caused by PQ treatment.

### 3.2. Effects of KL on Cytokine Changes after PQ Exposure

**Figure 2:**
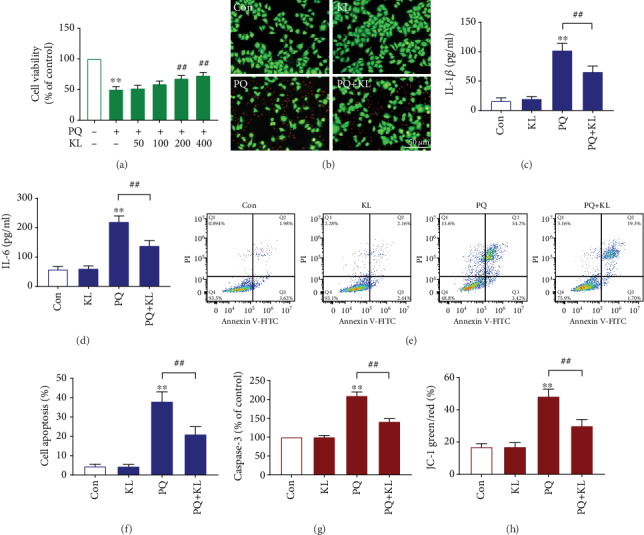
KL treatment improves cell viability, alleviates inflammatory response, and reduces apoptosis in PQ-exposed A549 cells. (a) A549 cell was preincubated with gradient concentrations of KL (50-400 pM) for 1 h then treated with or without 200 *μ*M of PQ for 24 h. Cell viability was measured using CCK-8 assay. (b) Fluorescence image of Calcein-AM/PI costained A549 cells receiving corresponding treatments. The levels of the proinflammatory cytokines IL-1*β* (c) and IL-6 (d) in the culture supernatants were determined by ELISA. (e) Apoptosis ratio of A549 cells receiving different treatments. Apoptosis was detected using flow cytometry, followed by Annexin V-FITC/PI double staining. (f) Comparisons of apoptosis ratio of A549 cells. The apoptosis ratio was calculated including early and late apoptosis. (g) The activity of caspase-3 in cell lysates was tested by a caspase-3 activity kit. (h) JC-1 staining monitors the changes in *ΔΨ*m, and the ratios of green/red fluorescence intensity indicate the mitochondrial depolarization. Data are shown as the mean ± SD. ^∗∗^*P* < 0.01 versus control group; ^##^*P* < 0.01 versus PQ group, ANOVA.

The contents of the proinflammatory cytokines IL-1*β* and IL-6 in the BALF and culture supernatants were measured by ELISA. After PQ treatment, the contents of IL-1*β* and IL-6 in the BALF or culture supernatants were obviously elevated compared with the other groups (Figures 1(b) and 1(c); Figures 2(c) and 2(d)). However, KL administration led to a significant reduction of IL-1*β* or IL-6 in the BALF and culture supernatants. In addition, there was no significant difference between the KL group and the control group for either cytokines. The findings suggest that KL exert an important effect on the suppression of inflammatory cascade induced by PQ.

### 3.3. KL Administration Reduced Lung W/D Ratio

The lung W/D ratio was measured to determine lung edema. As shown in Figure 1(d), after PQ treatment, the lung W/D ratio was increased approximately 2-fold compared with the control group. Furthermore, KL administration reduced the enhanced lung W/D ratio by 37.1% compared to PQ-treated mice.

### 3.4. Pathological Changes of PQ-Induced ALI *In Vivo*

H&E staining was performed to assess the impact of KL on PQ-induced lung injuries. As shown in Figure 1(e), lung tissues of mice from the KL group and the control group exhibited a normal lung structure, without signs of alveolar edema, alveolar collapse, or inflammatory cell infiltration. In contrast, mice in the PQ group presented severe degree of lung injury with inflammatory cell infiltration, exudation in alveolar cavity, alveolar septum thickening, alveolar hemorrhage, and alveolar architecture destruction. Compared with the PQ group, the above pathological changes in the PQ+KL group were significantly attenuated. Based on lung injury scores (Figure 1(f)), the mean value of the PQ+KL group reduced by 40.6% compared with that of the PQ group.

### 3.5. KL Administration Reduced Apoptosis *In Situ* of the ALI Mouse Model

To investigate the impact of KL on PQ-caused cytotoxicity, TUNEL assay was used to detect apoptosis in lung tissues. The experimental results in Figure 1(g) and Fig. S3 indicated that after PQ treatment, the apoptosis *in situ* of lung cells was augmented significantly compared to the control group and other treatment groups. However, KL pretreatment decreased the apoptosis rate by 34.0% compared with the PQ group. The above results suggested that *in vivo* administration of KL has a protective effect in lung tissue after PQ administration.

### 3.6. KL Treatment Increases Cell Survival of PQ-Exposed A549 Cells

Initially, we determined the toxic effect of PQ against A549 cells. The WST-8 assay was used to test cell viability after various treatments. A549 cells were incubated with PQ at a concentration range up to 1000 *μ*M for 24, 48, and 72 h for cell viability determination. We established that the application of PQ had slight cytotoxicity on A549 cells at low concentrations (<20 *μ*M), while the cell viability was significantly inhibited at concentrations greater than 20 *μ*M at different incubation time points (Fig. S1). The results demonstrated that PQ decreased cell survival in a dose-dependent style.

To study the effects of KL on PQ-induced cytotoxicity, A549 cells were preincubated with different doses of KL followed by PQ exposure. The results (Figure 2(a)) demonstrated that KL treatment dose dependently enhanced the survival of A549 cells exposed to PQ. Based on current findings, a dose of 400 pM of KL was used in the subsequent experiments. The cytotoxicity of PQ on A549 cells was further evaluated via costaining with Calcein AM and PI. Calcein-AM/PI costaining also demonstrated that preincubation with KL alleviated PQ-induced cytotoxicity to A549 cells (Figure 2(b)), with an increase in the proportion of living cells.

### 3.7. Effects of KL on Cell Apoptosis after PQ Treatment

Apart from cell survival, we next evaluated the effects of KL on PQ-induced cell apoptosis by Annexin V-FITC/PI dual staining (Figure 2(e)). The percentages of early- and late-apoptosis cells in different samples were calculated via flow cytometry. 24 h after PQ treatment, the cell apoptosis rate of the PQ group was significantly higher than that of the control group, while the apoptosis rate among the PQ+KL group reduced by 44.7% compared with the PQ group (Figure 2(f)). No significant difference was observed in the apoptosis rate between the KL group and the control group.

To further study the effects of KL on PQ-induced apoptosis, we tested the activity of caspase-3 in A549 cells receiving corresponding treatment (Figure 2(g)). We found that the caspase-3 activity was notably risen in the PQ group compared to that of the control group, while the caspase-3 activity of the PQ+KL group reduced by 32.5% compared to that of the PQ group. No significant difference was found in the caspase-3 activity between the KL group and the control group.

### 3.8. KL Ameliorated PQ-Caused Mitochondrial Depolarization

Previous study showed that PQ poisoning could trigger mitochondria-dependent apoptosis signaling. To verify whether mitochondrial apoptosis participates in the protection of KL against PQ cytotoxicity, we assessed the *ΔΨ*m with JC-1 staining. Similar to the previous study [23], exposure to 200 *μ*M of PQ for 24 h significantly decreased *ΔΨ*m in A549 cells. However, preincubation with KL alleviated PQ-induced decline of *ΔΨ*m by 37.9% (Figure 2(h)). No significant difference was observed in the JC-1 green/red ratio between the KL group and the control group. Collectively, the above findings suggested that KL could inhibit mitochondria-dependent cell apoptosis caused by PQ.

### 3.9. KL Inhibits PQ-Caused Oxidative Stress in A549 Cells

**Figure 3:**
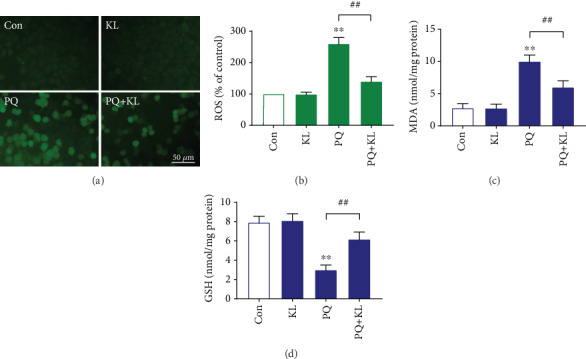
KL reduces intracellular ROS, lowers MDA, and improves GSH contents in PQ-exposed A549 cells. (a) The intracellular ROS production in A549 cells receiving different treatments was detected by the DCFH-DA method immediately after laser irradiation using a fluorescence microscope. (b) The ROS level of each sample was determined via DCF fluorescence intensity. The fluorescence intensity was calculated by Image-Pro Plus 6.0. The contents of MDA (c) and GSH (d) were measured by colorimetry as described in Materials and Methods. Data are shown as the mean ± SD. ^∗∗^*P* < 0.01 versus control group; ^##^*P* < 0.01 versus PQ group, ANOVA.

Previous studies have indicated that cell toxicity of PQ is related to ROS generation [3]. To determine whether KL administration improves cell survival by affecting the production of ROS, we compared the intracellular ROS generation abilities in different treatment groups using the DCFH-DA method. As presented in Figure 3(a), negligible fluorescence signal could be viewed in the KL group and the control group. After exposing to 200 *μ*M of PQ for 24 h, the ROS contents in A549 cells increased by about 1.5-fold compared to control (Figure 3(b)). KL incubation lowered PQ-induced intracellular ROS levels by 46.2%, indicating KL could reduce PQ-caused high level of ROS. To clarify the impact of KL on PQ-caused oxidative stress, we further tested the contents of MDA and GSH in A549 cells receiving corresponding treatments. The results showed that PQ exposure significantly caused an increase of MDA contents (Figure 3(c)) and a decrease of GSH contents (Figure 3(d)) when compared to control A549 cells. Notably, compared with PQ-exposed cells, KL preincubation decreased the elevated MDA contents by 40.0% and recovered the GSH contents by about 1-fold, respectively. These results demonstrated that KL administration was capable of ameliorating oxidative stress of PQ-exposed A549 cells.

### 3.10. Role of P38 MAPK Signal Molecule in Regulating Inflammation and Apoptosis in A549 Cells

**Figure 4:**
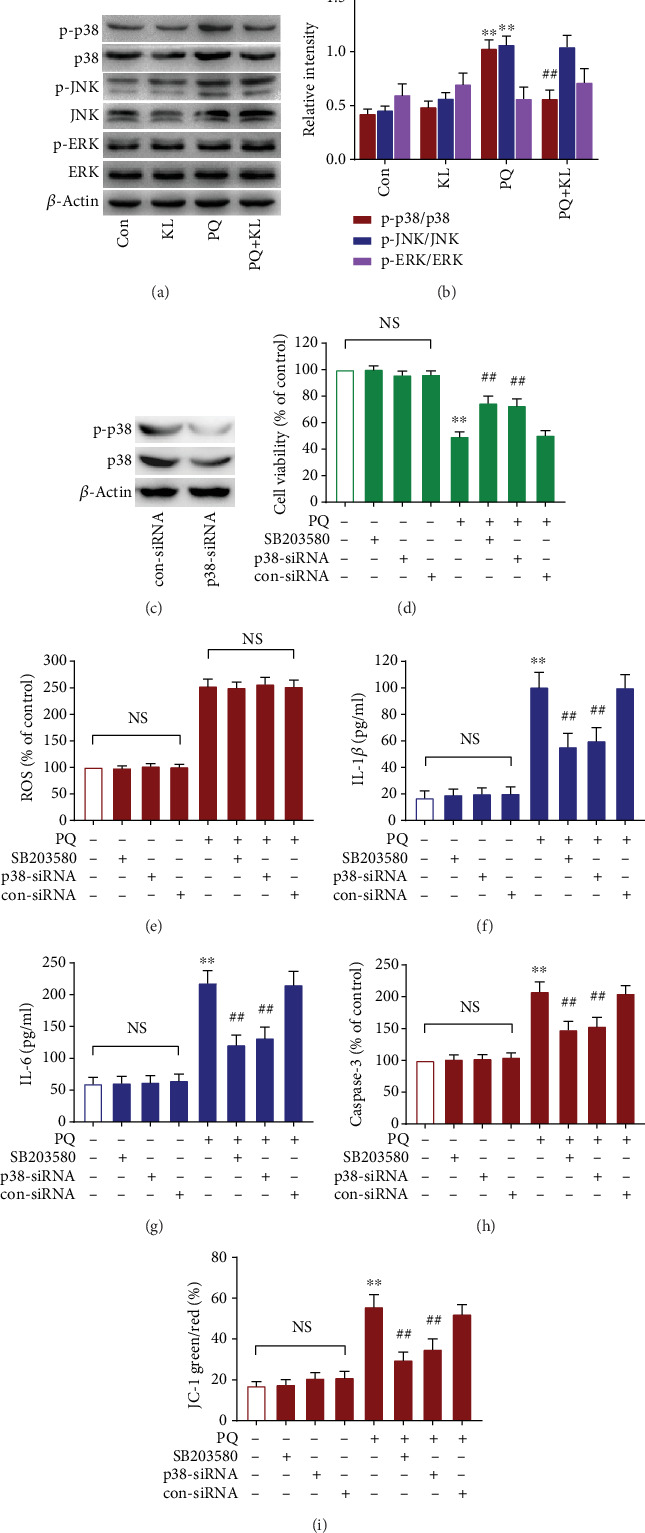
KL exerts protective roles on PQ-treated A549 cells by inhibiting ROS/P38 MAPK signaling. (a) The expression of P38 MAPK, JNK, and ERK was analyzed by western blotting. (b) Quantification of relative intensity of p-P38 MAPK, p-JNK, and p-ERK protein bands for each group. (c) Knockdown of P38 MAPK by siRNA was confirmed by western blot analysis. A549 cells were preincubated with P38-MAPK siRNA (50 nM) or SB203580 (10 *μ*M), subsequently treated with or without 200 *μ*M of PQ for 24 h. Cell viability (d) was measured using CCK-8 assay, and ROS production (e) was tested by DCFH-DA staining. The contents of IL-1*β* (f) and IL-6 (g) in the culture supernatants were evaluated by ELISA. The activity of caspase-3 (h) was detected by the caspase-3 activity kit, and the changes in *ΔΨ*m (i) were detected by JC-1 staining.

MAPKs can be activated by multiple extracellular stimuli and participate in many physiological processes, such as transformation, inflammation, and apoptosis. To determine whether MAPKs are involved in the signal transduction of KL attenuating PQ-caused ALI, the total or activated form of p38-MAPK, JNK, or ERK was detected by western blot. In contrast to control, the ratio of phosphorylated form to total form of p38-MAPK in A549 cells was raised by about 1.5-fold post PQ challenge (Figures 4(a) and 4(b)). Preincubation with KL downregulated the content of p38-MAPK phosphorylation by 45.2% in A549 cells receiving PQ treatment for 24 h. Likewise, the phosphorylated portion of JNK increased over 1.0-fold in PQ-exposed cells compared to control. However, the proportion of phosphorylated JNK was not significantly changed by KL pretreatment. Furthermore, the expression of phosphorylation of ERK did not significantly alter after PQ challenge or ghrelin administration.

**Figure 5:**
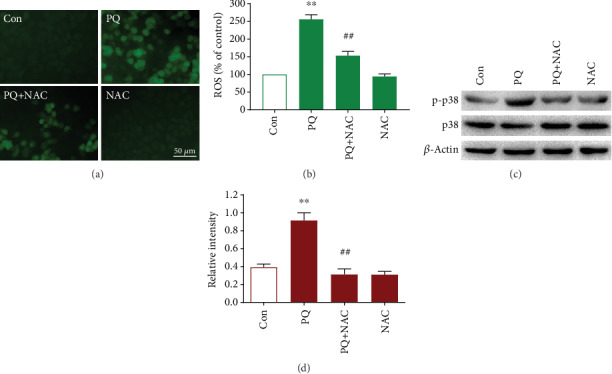
NAC treatment reduces intracellular ROS level and P38 MAPK activation in PQ-exposed A549 cells. (a) ROS production in A549 cells was measured by DCFH-DA staining under a fluorescence microscope. (b) Quantification of the DCF fluorescence intensity was conducted by Image-Pro Plus software. The expression of phosphorylated P38 MAPK was detected by western blotting (c) and quantified by the relative intensity of protein bands (d).

To investigate the molecular mechanism of KL attenuating PQ-caused ALI, SB203580 and p38 MAPK targeting siRNA were used to block p38 MAPK expression before exposed to PQ (Figure 4(c)). We found that downregulation of p38-MAPK expression enhanced the cell viability significantly compared with exposure to PQ alone (Figure 4(d)). However, the depletion of p38-MAPK had no significant effects on PQ-induced ROS overproduction (Figure 4(e)). We further found that downregulation of p38-MAPK significantly relieved PQ-caused elevation of IL-1*β* and IL-6 (Figures 4(f) and 4(g)), enhanced *ΔΨ*m, and reduced the elevation of caspase-3 (Figures 4(h) and 4(i)). The above findings showed that KL can alleviate PQ-induced inflammation and apoptosis by regulating ROS generation and p38-MAPK expression. To further explore the correlation between p38-MAPK signaling and oxidative stress, we utilized a classic ROS scavenger NAC to investigate the role of ROS in modulating the p38-MAPK signal pathway. Figures 5(a)–5(d) showed that, by contrast with PQ-exposed cells, NAC preincubation significantly reduced ROS generation and downregulated p38-MAPK phosphorylation.

## 4. Discussion

Lung tissue is the primary target organ during PQ intoxication. Alveolar epithelial cells can actively absorb and concentrate PQ distributed in the blood circulation through the polyamine transport system. PQ enrichment in lung tissue rapidly leads to the occurrence of ALI and the development of respiratory failure [24]. Due to the lack of special antidote, the mortality of PQ intoxication remains relatively high. In the current work, we proved for the first time that KL could attenuate the pathophysiological process of ALI caused by PQ via inhibiting oxidative stress in lung epithelial cells. Furthermore, we also found that the mechanism by which KL plays a vital role in relieving PQ-caused ALI is achieved by inhibiting ROS production in pulmonary epithelial cells.

Former studies demonstrated that oxidative stress exerts important roles on the pathogenesis of PQ-caused ALI [3]. The enrichment of PQ in lung tissues could induce the excessive production of ROS, causing oxidative injuries and inflammatory reactions in the lung tissue [25]. Intracellular PQ forms its free radical via NADPH-dependent reduction and oxidizes back to PQ cation by reacting with molecular oxygen. These reactions induce the production of ROS such as hydroxyl radical (^·^OH), hydrogen peroxide (H_2_O_2_), and superoxide anion (O_2_^·-^) [26]. The overproduction of ROS can lead to cellular component injuries by inducing oxidative damages to nucleic acid, proteins, and membrane fatty acids. Since excessive ROS generation proved to be the primary cause of PQ-caused ALI, inhibition of ROS production could be an effective way to treat PQ-induced ALI.

KL was originally identified in mice as a senescence suppressor gene, which could suppress multiple aging phenotypes and extend lifespan when overexpressed [27]. Recent studies have shown that KL protein can function as humoral factors and influence the pathophysiological process through modulating intracellular signal pathways [28]. The antiaging functions of KL closely associate with its antioxidative stress effect, which not only inhibits ROS production but also promotes ROS clearance. Previous findings showed that increased oxidative stress could inhibit KL expression while the downregulation of KL expression in tissues is prone to aggravate oxidative damage [29, 30]. Recent studies have revealed that overexpression of KL could alleviate oxidative stress-mediated cell injury [31, 32]. KL could eliminate excessive accumulation of ROS in several kinds of cells, including cardiomyocytes [33], vascular smooth muscle cells [34], and vascular endothelial cells [35], alleviating cellular damage by reducing elevated ROS levels.

Suga et al. reported that KL mutation in a mouse model could lead to the destruction of alveolar wall structure and cause pathological changes similar to emphysema [36]. Some studies recently reported that KL exerts significant effects on the pathogenesis of pulmonary fibrosis [37]. According to the above findings, we suggest that KL may regulate the oxidative stress state of pulmonary epithelial cells and thus might have therapeutic effect on the PQ-induced ALI. In this study, we found that KL treatment significantly attenuated acute pneumonocyte damages both *in vivo* and *in vitro*. To elucidate the impact of KL on the oxidative stress status induced by PQ, the MDA and ROS contents were detected in this work. We demonstrated that the MDA and ROS contents increased significantly after PQ exposure, and KL administration greatly reduced the PQ-caused expression of MDA and ROS following exposure to PQ. These findings indicated that oxidative stress arises after PQ exposure, which has a detrimental effect on intracellular oxidative homeostasis. Moreover, our findings indicated that treatment with KL exerted a protective role against PQ-caused ALI via regulating oxidative stress status in pulmonary epithelial cells.

ROS can activate and recruit inflammatory cells, facilitate inflammatory response, and promote the excessive release of additional ROS [38]. Previous reports indicated that inflammation response had a close relationship with PQ-induced lung damage [39]. After PQ exposure, the level of proinflammatory factors, such as IL-1*β*, TNF-*α*, IL-10, and TGF-*β* significantly elevated due to oxidation-antioxidation imbalance [25]. Among these cytokines, IL-1*β*, TNF-*α*, and IL-6 are regarded as significant proinflammatory mediators in the early stages of ALI, which plays significant roles in the occurrence of pulmonary inflammation [40]. The uncontrolled release of inflammatory factors aggravates the inflammatory response and accelerates tissue and organ damage. In accordance with these findings, we observed in our current research that IL-1*β* and IL-6 contents were notably increased in PQ-treated A549 cells and in the BALF of the PQ-induced ALI mouse model. Furthermore, our study demonstrated that KL administration could remarkably downregulate IL-1*β* and IL-6 expression levels following PQ treatment both *in vivo* and *in vitro*. The results presented here suggested the initiation of inflammatory reactions in pneumocyte following PQ poisoning, while administration with KL could alleviate ALI through inhibiting inflammatory response. Those results indicate that KL has the ability to attenuate PQ-caused ALI through suppression of inflammation.

Previous reports showed that excessive intracellular ROS can cause oxidative damage to membrane proteins and mitochondrial DNA, leading to structural changes and mitochondrial impairments [41]. Moreover, ROS-induced mitochondrial dysfunction, including oxidative injury itself, could lead to the aggravating imbalance between ROS generation and elimination, causing ROS overload and ultimately triggering the initiation of apoptosis [42]. The activation of apoptosis is characterized by the decline of the *ΔΨ*m and the presence of caspase activity. Among the caspase family, caspase-3 is one of the most important caspases and plays a vital role in mediating apoptosis. As shown in the present study, a reduced *ΔΨ*m and an elevated caspase-3 activity were noted upon PQ challenge, suggesting a possible role for apoptosis in PQ-induced anomalies. The present work also showed that the administration of KL could improve the *ΔΨ*m and reduce the activity of caspase-3 in A549 cells after exposure to PQ. Furthermore, the TUNEL method was used to detect apoptotic cells in lung tissues of the ALI mouse model. The *in vivo* experimental findings indicated that KL administration significantly alleviated PQ-induced apoptosis. Based on the above findings, we suggest that KL exerts antiapoptosis function via the mitochondria-dependent apoptosis pathway as KL administration could improve *ΔΨ*m and inhibit caspase-3 activation.

Our study on the anti-inflammatory and antiapoptosis mechanisms of KL demonstrated that KL treatment could inhibit P38-MAPK activation post PQ exposure. It is reported that MAPKs perform a significant function in regulating oxidative stress-related signaling and participate in the pathogenesis of PQ-induced ALI [43]. MAPKs are involved in regulating a variety of physiological processes in cells, including proliferation, differentiation, and apoptosis. We found in the present study that the phosphorylated form of P38-MAPK or JNK was significantly increased in A549 cells after PQ treatment, while KL treatment could reduce the elevated phosphorylation ratio of P38-MAPK after PQ exposure. The present results revealed that p38-MAPK activation performs important functions in the inflammation and apoptotic signaling pathways of A549 cells because downregulation of p38 MAPK using SB203580 or siRNA obviously improved cell viability, suppressed the contents of inflammatory cytokines, improved *ΔΨ*m, and reduced the activity of caspase-3. Previous findings revealed that oxidative stress-responsive MAPK signaling plays important roles in mediating inflammation and mitochondrial apoptosis [44]. This study demonstrated that KL could inhibit PQ-caused P38-MAPK activation in pneumocytes, which subsequently suppressed the inflammatory response and mitochondrial apoptosis.

Our results further showed that pretreatment with the ROS scavenger NAC contributed to the inhibition of ROS production post PQ challenge while abolished PQ-induced P38-MAPK activation. In addition, this study further revealed that pretreatment with SB203580 or p38 MAPK siRNA had no significant effect on intracellular ROS production post PQ challenge, which supports the hypothesis that ROS molecules acts as an upstream regulator of P38-MAPK. Former studies have shown that p38 MAPK activation was downstream to ROS generation [45], which was consistent with our current research. Based on the above results, we believe that KL plays important roles in the activation of downstream p38-MAPK signal pathway to regulate inflammation and mitochondrial apoptosis after PQ exposure. Based on the above results, we believe that KL-induced inhibition of ROS production post PQ exposure exerts important roles in activating downstream P38-MAPK signaling pathways to regulate inflammation and mitochondrial apoptosis (Fig. S2).

In conclusion, we performed *in vivo* and *in vitro* studies to assess the effects of KL administration on ALI induced by PQ. We demonstrated that KL had a protective effect on pulmonary epithelial cell damage and significantly improved survival rate and alleviated lung tissue injury. The mechanism of KL may lie in the inhibition of oxidative stress-mediated inflammatory responses and the reduction in pulmonary epithelial cell apoptosis. Our research provides new insights into the pathogenesis and treatment of PQ-induced ALI; however, the detailed molecular mechanisms deserve further investigation in the future.

## Data Availability

The data used to support the findings of this study are included within the article.

## Supplementary Materials

Supplementary Materials Figure S1: PQ treatment deceased the A549 cell viability in a dose- and time-dependent manner. Figure S2: schematic illustration. Schematic illustration showing klotho alleviates paraquat-induced lung injury through inhibiting ROS/P38 MAPK signaling-regulated inflammatory responses and mitochondrial apoptosis. Figure S3: effects of KL administration on PQ-induced apoptosis of lung cells. The representative images of apoptosis in situ. Apoptosis was measured using TUNEL assay.
