# Supplementary material for: Klotho Alleviates Lung Injury Caused by Paraquat via Suppressing ROS/P38 MAPK-Regulated Inflammatory Responses and Apoptosis
Source: Oxid Med Cell Longev. 2020 May 13;2020:1854206. doi: 10.1155/2020/1854206 (PMC7244968; doi:10.1155/2020/1854206)
Supplement: Supplementary Materials — Figure S1: PQ treatment deceased the A549 cell viability in a dose- and time-dependent manner. Figure S2: schematic illustration. Schematic illustration showing klotho alleviates paraquat-induced lung injury through inhibiting ROS/P38 MAPK signaling-regulated inflammatory responses and mitochondrial apoptosis. Figure S3: effects of KL administration on PQ-induced apoptosis of lung cells. The representative images of apoptosis in situ. Apoptosis was measured using TUNEL assay. [file 1854206.f1.pdf]

Supplementary Materials

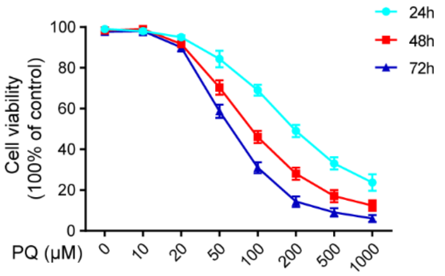

Fig. S1 PQ treatment deceased the A549 cell viability in a dose- and time-dependent manner. A549 cells were incubated with PQ at a concentration range up to 1000 μM for 24, 48, and 72 h, then cell viability was determined by the WST-8 assay.

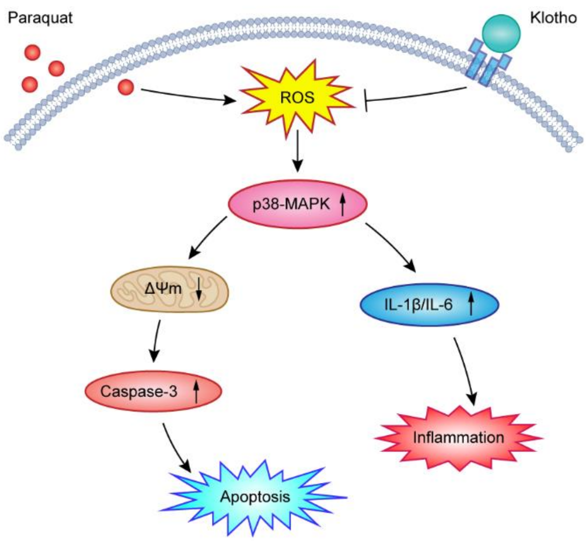

Fig. S2 Schematic illustration. Schematic illustration showing klotho alleviates paraquat-induced lung injury through inhibiting ROS/P38 MAPK signaling-regulated inflammatory responses and mitochondrial apoptosis.

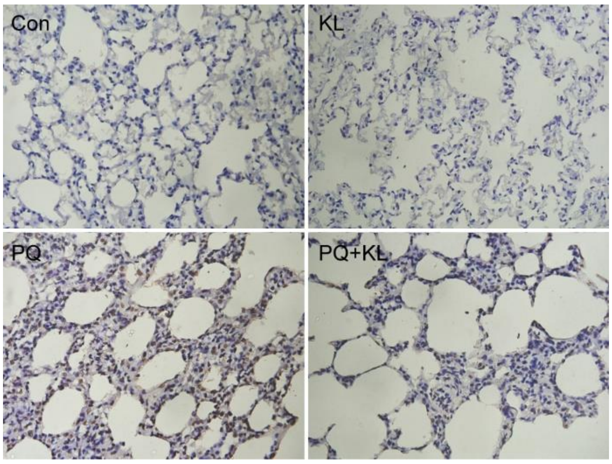

Fig. S3 Effects of KL administration on PQ-induced apoptosis of lung cells. The representative images of apoptosis in situ. Apoptosis was measured using TUNEL assay. **Fig. S3 Effects of KL administration on PQ-induced apoptosis of lung cells.** The representative images of apoptosis in situ. Apoptosis was measured using TUNEL assay.
